# Elevated T/E_2_ Ratio Is Associated with an Increased Risk of Cerebrovascular Disease in Elderly Men

**DOI:** 10.1371/journal.pone.0061598

**Published:** 2013-04-24

**Authors:** Yanping Gong, Haiying Xiao, Chunlin Li, Jie Bai, Xiaoling Cheng, Mengmeng Jin, Boruo Sun, Yanhui Lu, Yinghong Shao, Hui Tian

**Affiliations:** 1 Department of Geriatric Endocrinology, The Chinese People's Liberation Army General Hospital, Beijing, China; 2 Department of Geriatric Clinical Laboratory, The Chinese People's Liberation Army General Hospital, Beijing, China; Clermont Université, France

## Abstract

**Objective:**

To investigate the relationship between sex hormones and the risk of vascular disease in elderly men and to evaluate the advantages and disadvantages of testosterone replacement.

**Methods:**

A total of 337 men, aged 60 to 91 years, were enrolled in this single-center, cross-sectional study, and their sex hormone levels were assessed. Linear and logistic regression analyses were utilized to compare the sex hormone levels between patients with and without vascular disease. The nonparametric K-sample test was used for inter-group comparisons.

**Results:**

Aging and abnormal metabolism were both significantly associated with an increased risk of vascular diseases and changes in sex hormone levels. Primary linear and logistic regression analyses showed no significant differences in sex hormone concentrations between patients with and without vascular diseases after adjusting for age. Logistic regression with abnormal metabolism as categorical variable showed that free testosterone (FT) and free estradiol (FE_2_) had significant relationships with CEVD risk (P<0.05). In further regression with all metabolic continuous variables included, the testosterone/estradiol (T/E_2_) ratio replaced FT and FE_2_ (P<0.05). Trend line analyses showed that T/E_2_ actually had a binomial linear correlation with the risk of cerebrovascular disease; its best protective effect occurred at values of 0.13–0.15, with an OR value extremely close to those of FT and FE_2_ (0.23 vs. 0.24–0.25).

**Conclusion:**

T/E_2_ balance plays a key role in the relationship between sex hormones and the risk of cerebrovascular disease. The balance between T and E_2_ may be more important than their absolute quantities. Extremely low T/E_2_ and inappropriately high T/E_2_ ratio can both harm the brain blood vessels. Careful consideration should be given before beginning testosterone replacement treatment, and supplementing with estrogen seems to be a good way to protect blood vessels of the brain in elderly men.

## Introduction

The vasculature is a target tissue for sex steroid hormones. Estrogen and Testosterone (TT) have become research hotspots in recent years [1]–[3]. The relationship between TT and vascular disease has been the subject of much research. However, there is intense debate regarding the role of TT in vascular function and cardiovascular disease (CVD) [3]. Low TT is associated with the progression of atherosclerosis, the production of proinflammatory cytokines, increased arterial thickness, and increased glucose, total cholesterol and low-density lipoprotein, all of which contribute to CVD [4], [5]. However, high TT has been associated with sudden cardiac death, liver disease and suicidal behavior [3], [6]. Being male is a risk factor for CVD, as males have an earlier occurrence and higher prevalence of many cardiovascular and metabolic-related diseases [7].

In addition to TT, estrogen is significantly associated with the risk of vascular disease. Menopausal women have much lower incidences of heart and renal disease compared with men of the same age, but this sex difference in favor of women gradually disappears after menopause; indeed, the cardiovascular risk becomes even higher in older women [2], [7]. The recent Nurse's Health Study [8], WISE Study [9], and the Women's Health Initiative [10] have demonstrated that early menopause in young women due to ovarian dysfunction or bilateral oophorectomy is associated with an increased risk of CVD compared with women exhibiting normal endogenous estrogen levels. Endogenous estrogen may be cardioprotective in men as well [11], [12]. In men, significant amounts of estrogen can be produced by the enzyme aromatase, which converts C19 androgenic steroids into 17β-estradiol (E_2_). In healthy young men, aromatase inhibition lowers plasma E_2_, and this decrease is associated with decreased flow-mediated dilatation of the brachial artery [13].

In contrast to women, whose estrogen levels drop precipitously at menopause, men of all ethnicities undergo a gradual decline in TT. Up to 30% of men over 60 years of age have TT levels that are below the normal range for young men; a 0.8% per year decrease in total TT and a 2% per year decrease in bioavailable TT was reported in a cross-sectional study of elderly men [14], [15]. Elderly men, who suffer from many aging symptoms, such as fatigue and loss of muscle mass, could benefit greatly from testosterone replacement. Although much is known about the effects of hormones on blood vessels, there is still much to be discovered. It is necessary to determine the side effects of hormones on vessels. We designed this study to investigate the relationship between the changes in male sex hormone levels and the risk of vascular disease in elderly men.

## Methods

### Study population

This single-center, cross-sectional study enrolled men above 60 years of age. We screened 1920 men who were between 60 and 91 years of age who had routine physical examinations at the Chinese People's Liberation Army General Hospital between May and July 2011. Potential subjects were from all 16 districts and both counties of Beijing. After excluding those who had a malignancy or a disease that was treated using TT or corticosteroids, 508 elderly men (26.5%) whose endogenous sex hormone levels had been determined remained as potential subjects. Among these potential subjects, the following patients were excluded: 90 who did not undergo biochemical examination, 79 who suffered from kidney dysfunction (Cr >113 µmol/L), 1 who was diagnosed with hyperparathyroidism and 1 who had moderate anemia. No patient was excluded based on a diagnosis of liver dysfunction (alanine aminotransferase or aspartate aminotransferase 3 times above the normal level). Thus, 337 subjects (69.4% from 558) were included in the analyses. The diagnosis of coronary heart disease or cerebrovascular disease was confirmed by reviewing each patient's medical record. The coronary heart diseases included angina, silent (asymptomatic) myocardial ischemia and myocardial infarction. The diagnoses of cerebrovascular disease included transient cerebral ischemia, vertebrobasilar insufficiency, cerebral thrombosis, cerebral infarction and cerebral hemorrhage.

### Clinical parameters

After the patient fasted for at least 8 hours overnight, baseline parameters were obtained by trained physicians the next morning between 6∶00 and 8∶00. The recorded baseline parameters included height, weight, BMI (kg/m^2^), age, sex, medical history, medication use and relevant diseases. Blood pressure (BP) was measured twice at the right brachial artery with the patient in the sitting position after a 5 minutes rest using the semi-automatic oscillometric method.

### Sex hormone measurements

A fasting venous blood sample was obtained in the morning between 6∶00 and 8∶00 after an overnight fast. A postprandial venous blood sample was extracted two hours after eating 100 g of carbohydrates or drinking liquid containing 75 g of glucose. The blood sample was immediately centrifuged for 15 min at 4°C, and the platelet-free serum and plasma were stored at −20°C. All of the samples were tested within 1 week. Total TT, E_2_, sex hormone binding globulin (SHBG), follicle-stimulating hormone (FSH) and luteinizing hormone (LH) were measured using an electrochemiluminescence system automatically (Roche Cobas C6000, Roche Diagnostics GmbH, Mannheim, Germany). Free testosterone (FT) and bioactive testosterone (BT) were calculated using Vermeulen's formula, free estradiol (FE_2_) was calculated according to the formula reported by Sabina Rinald et al [16], and the T/E_2_ and E_2_/SHBG ratios were also calculated. The FT index (FTI) was calculated as FTI  =  T/SHBG, and the testosterone secretion index (TSI) was calculated as TSI  =  T/LH.

### Other laboratory measurements

Biochemical indices were determined using serum samples that were collected after fasting, except for the postprandial blood glucose (PBG) measurement, which required postprandial serum samples. An automated enzymatic procedure (Cobas E601, Roche) was used to determine the fasting blood glucose (FBG) and PBG (Roche). High-density lipoprotein (HDL) and low-density lipoprotein (LDL) cholesterol, triglycerides (TG), serum total cholesterol (TC), uric acid (UA), blood urea nitrogen (BUN), creatinine (Cr), glutamic-pyruvic transaminase (ALT) and glutamic-oxaloacetic transaminase (AST) were similarly measured. Diagnoses were defined according to the case records at the corresponding medical institution, if treatments or medications were utilized. New diagnoses were made if abnormal results were obtained, as described below. Diabetes mellitus was defined as FBG >6.9 mmol/L and/or PBG >11.0 mmol/L and/or treatment with insulin or oral hypoglycemic agents. Impaired glucose tolerance (IGT) was defined as FBG ≤6.9 mmol/L and 7.8 mmol/L < PBG ≤11.0 mmol/L. Impaired fasting glucose (IFG) was defined as 6.1 mmol/L < FBG ≤6.9 mmol/L and PBG ≤7.8 mmol/L. Impaired glucose regulation (IGR) was defined as the combination of IGT and IFG. Hypertension was defined as systolic BP >140 mmHg and/or diastolic BP >90 mmHg and/or the use of anti-hypertensive medication. A patient was considered positive for hyperlipidemia if any lipid test result was abnormal (reference ranges defined by the Biochemistry Department of the Chinese People's Liberation Army General Hospital as TC >5.7 mmol/L, TG >1.7 mmol/L, HDL <1.6 mmol/L and LDL >3.4 mmol/L) and/or if the patient was taking any lipid-lowering medications. Hyperuricemia was defined as uric acid >420 μmol/L and/or if corresponding medications were used. A patient was determined to be overweight with a BMI >24 kg/m^2^ and obese with a BMI >28 kg/m^2^. Diagnostic criteria for metabolic syndrome proposed by the Chinese Diabetes Society (CDS) were used (see Figure S1).

### Statistical analyses

SPSS 17.0 (SPSS Inc, Chicago, IL, USA) was used for both data management and analyses. Continuous variables were presented as the mean ± SD. The distribution of data was evaluated before analyses. FSH, LH, E/SHBG, PROG and TSI did not have a normal distribution, exponential distribution or Poisson distribution. Therefore, we categorized the patients by vascular disease situation: cardiovascular disease only (CAVD), cerebrovascular disease only (CEVD), both type of vascular disease (TVD) and controls. After this categorization, only LH, FSH, and PROG did not have a normal distribution. Multiple-factor linear regression was used to compare normally distributed data, and nonparametric K-sample tests were used to compare non-normally distributed data. Age, smoke, all sex hormones, BMI, BP (SBP, DBP), plasma glucose (FBG, PBG), insulin resistance (insulin, C peptide and homeostasis model assessment-estimated insulin resistance (HOMA-IR)), plasma lipids (TC, LDL, HDL and TG), plasma uric acid, and abnormal metabolism (MS and number of abnormal metabolic test results) were included in the stepwise regression analyses of vascular diseases. Index and log conversion of LH, FSH, PROG did not change the explanation effect of the regression equation. Classifying variables were reported as percentages. A χ^2^ test was conducted for two-group comparisons. Nonparametric K-sample tests were used for inter-group comparisons. Trend line analysis was used to analyze the tendency of sex hormone levels to increase with age. The R^2^ value was used to evaluate the effectiveness of the trend line. For these analyses, P<0.05 was considered statistically significant. The 95% confidence interval (95% CI) was calculated to describe the magnitude of associations.

## Results

### Vascular disease in this population

**Figure 1:**
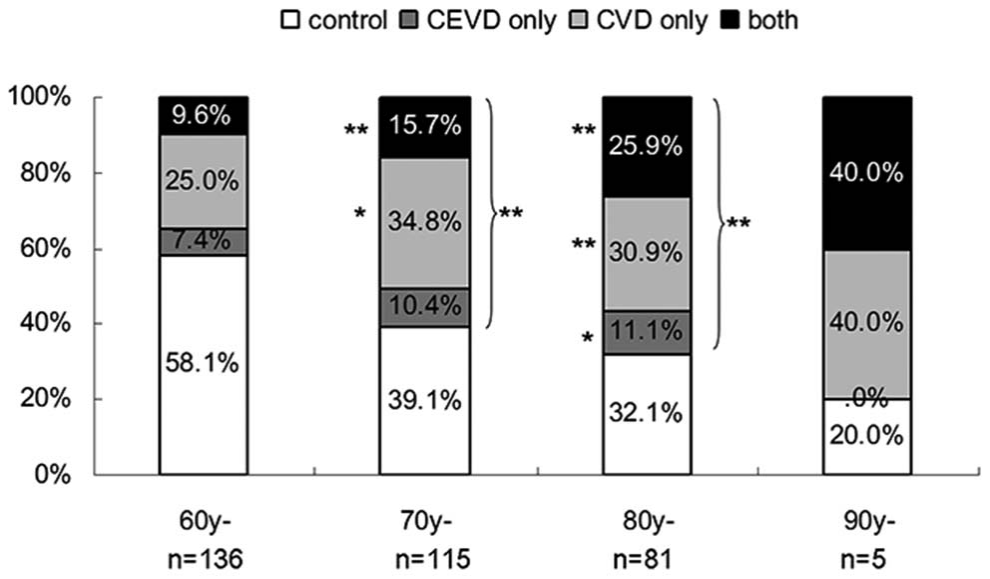
Age distribution of vascular diseases. The age range of the 60-year-old group was 60–69 years, of the 70-year-old age group was 70–79 years, of the 80-year-old group was 80–89 years and of the 90-year-old group was 90–91 years. The morbidity of CEVD, CAVD, TVD and EVD are shown as a percentage of each age group. * P<0.05, ** P<0.01 versus the morbidity of vascular diseases in the 60-year-old group.

A total of 337 subjects qualified for this study. The age ranged from 60 to 91 years. Included in the cohort were 101 (29.97%) and 31 (9.20%) subjects with CAVD and CEVD, respectively. There were 54 (16.02%) subjects with both vascular diseases (TVD), which meant 186 (55.19%) subjects had either type of the vascular diseases (EVD). The age distribution of the patients with vascular disease is depicted in Figure 1. The morbidity of cardiovascular disease, cerebrovascular disease and all vascular disease all increased with age (P<0.01).

### Sex hormone levels in this population

**Figure 2:**
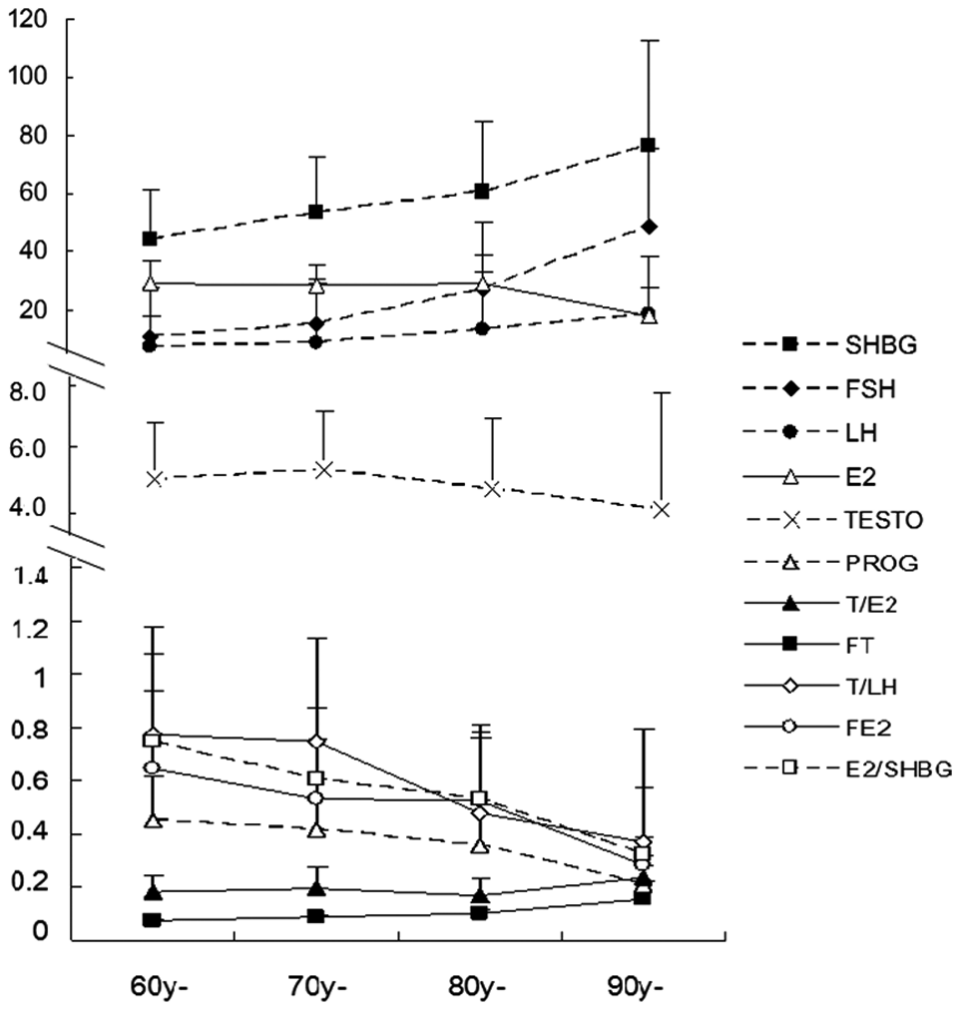
Changes in sex hormones by age category. The mean ± SD TT (nmol/L), FT (nmol/L), SHBG (nmol/L), E_2_ (pmol/L), FSH (IU/L), LH (IU/L), PROG (nmol/L), TSI (nmol/IU), FTI, T/E_2_ and E_2_/SHBG grouped by age are shown by curves. The age categories are the same as in Fig. 1. SHBG, FT and T/E_2_ increased with age (shown by filled symbols), but the others declined (shown with hollow symbols). FSH, LH, SHBG, FT, FTI, TSI, FE_2_, E_2_/SHBG and PROG had a linear relationship with age, and the R^2^ values of their linear equations were 0.92, 0.94, 0.97, 0.88, 0.99, 0.92, 0.85, 0.96 and 0.90. Significant differences between age groups (*P*<0.01) were observed in FSH, LH, SHBG, FTI, TSI, FE_2_, and E_2_/SHBG.

The sex hormone levels of this population are presented in Table 1, and the sex hormone levels categorized by age group are illustrated in Figure 2. Serum FSH, LH, SHBG, FT and T/E_2_ ratio increased with age, but the others declined. FSH, LH, SHBG, FT, FTI, TSI, FE_2_, E_2_/SHBG ratio and PROG were linearly correlated with age, with R^2^ values of the linear equation of 0.92, 0.94, 0.97, 0.88, 0.99, 0.92, 0.85, 0.96 and 0.90, respectively. FSH, LH, SHBG, FTI, TSI, FE_2_, and E_2_/SHBG changed significantly with age (*P*<0.01).

**Table 1:** Characteristics of the sex hormones in the study population.

	n	Mean ± SD	Range
FSH (IU/L)	336	16.89±16.89	0.10–119.40
LH (IU/L)	336	9.42±6.29	0.10–45.69
E_2_ (pmol/L)	335	28.51±8.6	5.00–55.90
E_2_/SHBG	335	0.64±0.00	0.14–1.76
FE_2_	335	0.59±0.27	0.17–1.88
PROG (nmol/L)	336	0.41±0.18	0.00–1.09
TT (nmol/L)	337	5.04±1.93	0.03–11.16
T/E_2_	335	0.18±0.00	0.00–0.44
FTI	337	0.10±0.03	0.00–0.22
TSI (nmol/IU)	336	0.69±0.39	0.00–2.26
FT (nmol/L)	337	0.22±0.08	0.00–0.67
BT (nmol/L)	337	5.80±1.91	0.01–11.1
PRL (μg/L)	336	28.51±8.60	0.00–55.90
SHBG (nmol/L)	337	51.82±21.42	11.38–172.50

### The association between vascular disease and sex hormone levels

As shown in Table 2, plasma TT, BT, FTI, TSI, E_2_, E_2_/SHBG and PCG were lower in patients with vascular disease compared with the control group, while LH, FSH and SHBG were higher in the vascular disease groups. The changes in the above sex hormones in CEVD were more obvious than in CAVD. LH, FSH, SHBG, FTI and TSI were significantly different between vascular disease groups. After adjustment for age and smoke, the gaps in these sex hormones between controls and vascular disease patients decreased, and all the significant differences disappeared. Logistic analyses showed the same results with age and smoke as covariant.

**Table 2:** The differences in sex hormone levels after grouping the subjects by vascular disease.

VD		TT	FT	BT	T/E_2_	E_2_	E_2_/SHBG	FE_2_	PROG	LH	FSH	FTI	TSI	SHBG
Control (n = 150)	A	5.19±1.83	0.22±0.08	5.15±1.81	0.19±0.06	29.24±8.45	0.68±0.32	0.60±0.28	274.45±166.13	8.45±4.90	13.84±14.19	0.11±0.03	0.76±0.41	49.64±21.58
	B	5.16±0.16	0.22±0.01	5.13±0.16	0.19±0.01	29.14±0.71	0.65±0.03	0.60±0.28	275.05±11.24	9.39±0.49	16.32±1.29	0.11±0.09	0.72±0.03	51.63±1.67
CEVD (n = 31)	A	5.02±1.90	0.25±0.14	4.97±1.89	0.19±0.09	27.23±7.97	0.58±0.29	0.50±0.21*	218.51±77.61	11.28±8.83*	21.65±22.15*	0.10±0.03*	0.64±0.42	55.32±22.32
	B	5.06±0.35	0.25±0.02	4.99±0.35	0.19±0.01	27.34±1.54	0.61±0.06	0.50±0.02	217.54±24.73	10.55±1.07	19.57±2.84	0.18±0.01	0.68±0.07	53.24±3.67
CAVD (n = 101)	A	5.19±1.82*	0.21±0.08*	4.96±1.87*	0.18±0.07	28.40±8.10	0.64±0.30	0.52±0.21	243.71±116.04	9.37±6.23	17.29±16.81	0.10±0.03*	0.66±0.35*	49.75±18.29
	B	4.94±0.19	0.21±0.01	4.94±0.19	0.18±0.01	28.44±0.84	0.65±0.03	0.51±0.02	242.96±13.44	9.17±0.58	17.01±1.54	0.11±0.00	0.68±0.04	49.03±2.01
TVD (n = 54)	A	5.03±2.02	0.21±0.09	4.99±1.99	0.18±0.08	28.51±8.87	0.58±0.30*	0.52±0.23	225.45±166.13*	10.89±6.64**	20.70±17.30**	0.09±0.03**	0.61±0.37*	57.86±22.66
	B	5.01±0.27	0.21±0.01	4.94±0.27	0.18±0.01	28.60±1.17	0.62±0.04	0.51±0.02	227.18±18.93	9.52±0.82	17.08±2.17	0.10±0.00	0.61±0.05	54.56±2.81
EVD (n = 183)	A	5.00±1.93	0.22±0.10	4.95±1.91	0.18±0.08	28.24±8.27	0.64±0.31*	0.52±0.22	234.34±101.73**	10.12±6.84*	18.98±17.91**	0.10±0.03**	0.64±0.37**	52.98±20.52
	B	4.98±0.14	0.22±0.01	4.95±0.14	0.18±0.01	28.31±0.63	0.63±0.02	0.51±0.02	234.47±10.12	9.49±0.44	17.45±1.16	0.10±0.00	0.67±0.03	51.26±1.51

VD: vascular diseases; CEVD: cerebrovascular disease; CAVD: cardiovascular disease; TVD: both CEVD and CAVD; EVD: more than one type of vascular diseases.

A represents the difference in sex hormone levels in patients with various vascular diseases without adjusting for age and smoke.

B represents the difference in sex hormone levels in patients with various vascular diseases after adjusting for age and smoke.

Considering that abnormal metabolism also had a close correlation with vascular disease, we performed logistic regression with metabolic indexes as covariates (see Table 3). In regression with categorical variables of abnormal metabolism as covariates, PROG, FT, FE_2_ and MetS showed close correlations with cerebrovascular disease. The value of exp(B) for these four parameters was 1.00, >1000, 0 and 4.24, respectively. In the regression including continuous variables of metabolic parameters as covariates (see Table 3), the significant correlations of FT and FE_2_ with cerebrovascular disease disappeared, and instead, elevated T/E_2_ ratio, LH and C-peptide were correlated with increased incidence of cerebrovascular disease (P<0.05), with an exp(B) value of >1000, 1.1 and 4.09, respectively.

**Table 3:** Logistic regression analyses of cerebrovascular disease.

		PROG	FT	FE_2_	E_2_/SHBG	T/E_2_	LH	Age	MetS	C-peptide
model 1										
	sig	0.05	0.05							
	exp(B)	1	75.71							
	95%C.I.	0.99–1.02	1.10–>1000							
model 2										
	sig	0.06	0.09					0.05		
	exp(B)	1.0	45.16					1.058		
	95%C.I.	0.99–1.00	0.58–3494					1.01–1.12		
model 3										
	sig	0.05	0.02	0.04	0.58		0.11	0.43	0.022	
	exp(B)	1	>1000	0.00	166.65		1.07	1.03	4.24	
	95%C.I.	0.99 –1.00	4.17–>1000	0.00–0.69	0.84–>1000		0.99–1.15	0.97–1.11	1.23–14.67	
model 4										
	sig	0.07	0.41	0.106		0.05	0.03			0.03
	exp(B)	1	>1000	0.21		>1000	1.10			4.09
	95%C.I.	0.99 –1.00	<0.01–>1000	0.16–1.52		0.95–>1000	1.00–1.28			1.14–14.68

Model 1: all sex hormones were included in the regression equation.

Model 2: all sex hormones and age were included in the regression equation.

Model 3: all sex hormones, age, all metabolic categorical variables and hepatic/renal function were included in the regression equation.

Model 4: all sex hormones, age, all metabolic continuous variables and hepatic/renal function were included in the regression equation.

No correlations were found between sex hormones and CAVD, TVD, or EVD. Instead, abnormal glucose level, hyperlipidemia, BMI and MetS showed close correlations with these 3 vascular diseases.

### Trend line of FT, FE_2_, and T/E_2_ in elder men

**Figure 3:**
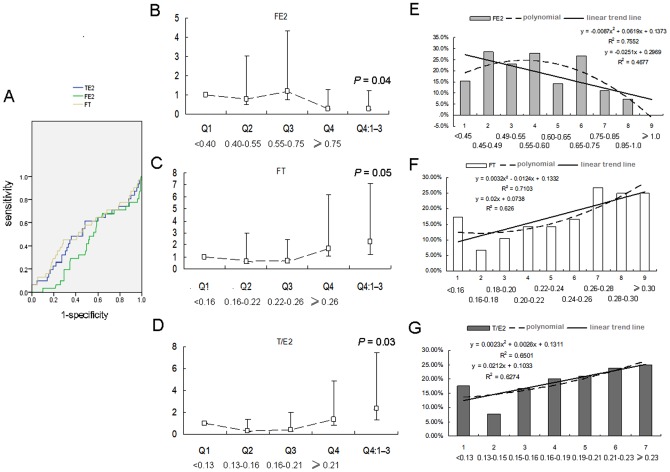
Trend lines of FT, FE_2_, and T/E_2_ and CEVD in elderly men. ROC curves of FT, FE_2_ and T/E_2_ are shown in 3A. Using men in quartile 1 (Q1) as a reference, the risk of CEVD of Q2, Q3, and Q4 are shown in Fig. 3B–D. “Q4:Q1–3” means the risk of CEVD of Q4 compared to the pool of Q1–3. We divided quartiles 2–4 of FE_2_, FT and T/E_2_ into 8, 8 and 6 groups by increments of 0.05 pmol/L, 0.02 nmol/L and 0.01 units, respectively (10 to 20 people per group). The trend lines and equations are shown in Fig. 3E–G.

ROC curves were first used to explore the possible cut-off values of FT, FE_2_ and T/E_2_, but the areas under the curve were not ideal (the highest was 0.59, from T/E_2_, see Figure 3A). This combined with the fact that the 95% CIs of FT, FE_2_ and T/E_2_ were not present in a relatively confined areas, we speculated that the relationships of FT, T/E_2_ and CEVD were not simple linear relationships. We analyzed these parameters based on quartiles, and binomial linear relationships of CEVD with these three sex hormones were found. Positive trends of FT and T/E_2_ and a negative trend of FE_2_ were detected, which were consistent with the values of exp(B) from logistic regression. FT and T/E_2_ in quartile 4 (Q4) were associated with a higher risk of a CEVD event (OR  = 2.23 and 2.36, P = 0.05 and 0.03, respectively), and FE_2_ in Q4 was associated with a lower risk of a CEVD event (OR  = 0.29, P = 0.04), compared with the values in Q1–3 (Fig. 3B–D).

We further divided quartiles 2–4 of FE_2_, FT and T/E_2_ into 8, 8 and 6 groups by increments of 0.05 pmol/L, 0.02 nmol/L and 0.01 units, respectively, creating subgroups of 10–20 people each. The trend lines and equations are shown in Fig. 3E–G. Calculated from the formulas, the best protective effect was observed when FE_2_ increased to more than 0.65–0.75 pmol/L, FT remained at 0.16–0.18 nmol/L, and T/E_2_ remained at 0.13–0.15. The OR values of these areaes compared with the highest risk areas of FE_2_, FT and T/E_2_ were extremely close to (0.24, 0.25 and 0.23, respectively).

## Discussion

In this study population, age is an important factor that influences both vascular diseases and sex hormones: the incidence of vascular disease increases with age, and the levels of sex hormones vary with age. However, age is not the only factor that influenced sex hormones. Our earlier study confirmed that abnormal metabolism can change the sex hormone levels in elder men [17]. FT and FE_2_ were correlated with CEVD in linear regressions with categorical variables of abnormal metabolism included, and the effect of elevated T/E_2_ was highlighted in linear regressions with continuous variables included, though no significant differences in sex hormones could be found with only age adjustment in linear and logistic regression analyses.

The changes in testosterone and estrogen levels with increasing age are different between men and women [18], [19]. Testosterone peaks in men are in young adulthood. Starting around age 30, however, testosterone levels gradually decline. One reason for this change is that testosterone is more likely to be converted to estradiol by aromatase in aging men. Furthermore, as the ratio of testosterone to estrogen begins to shift, the chance that estrogen will bind to androgen receptors (normally used by testosterone) increases. The body then responds by producing less testosterone, which shifts the balance even more.

Previous cohort studies of the association between serum testosterone and CV events have found contradictory results. Large population-based prospective studies reported an inverse association (Rancho Bernardo [20], European Investigation into Cancer-Norfolk [21]), no association (Massachusetts Male Aging Study [15], National Health And Nutrition Examination Survey [22]), or a positive association (Vlachopoulos [6], [15], Wittnich [23]) between total testosterone and CV disease mortality [15], [24]. Our study showed that the trend line of CEVD and finely grouped FT values was defined by a binomial line, and maintaining a proper range of FT (0.16–0.18 pmol/L) might be helpful to prevent vascular diseases. Considering that this range of FT was lower than that observed in most of our patients, higher FT would usually have positive relationship with increasing risk of CEVD. Subjects in the highest quartile of FT had a 2-fold higher risk of CEVD compared with the lowest quartile. At the same time, the risk of cerebrovascular disease could be increased by androgen supplementation, so careful consideration should be given before beginning treatment.

Estrogen can also offer vascular protection [2], [12], [13], [18]. Our study confirmed that higher estrogen significantly decreased the VECD incidence, which was only 29% in the highest FE_2_ quartile compared with the lowest quartile. At the same time, our data suggest that 7.5 pmol/L of free estrogen is the cut-off point above which the risk of CEVD decreases 86% compared to the risk of the highest-risk group. Supplementation with estrogen therefore seems a good way to protect the blood vessels of the brain in elderly men. However, for elderly men whose estrogen is already extremely high, simple estrogen replacement might not be appropriate. A recent study [25] measured blood estradiol in 501 men with chronic heart failure and found that men in the highest quintile of estradiol (serum estradiol more than 37.40 pg/mL or 137.26 pmol/L) had an increased death rate. The authors recommended that in aging males, estradiol should be kept under 30 pg/mL (110.1 pmol/L). Neither E_2_ nor FE_2_ in our study reached such a high level, so we did not find an upper recommended limit of FE_2_. However, when metabolic abnormalities were set as continuous variables in the logistic regression analyses, the effect of the T/E_2_ ratio gained significance. This meant FE_2_ could not increase indefinitely along with lower T in elderly men, and the upper the limit of FE_2_ depended on the ratio of T/E_2_.

**Figure 4:**
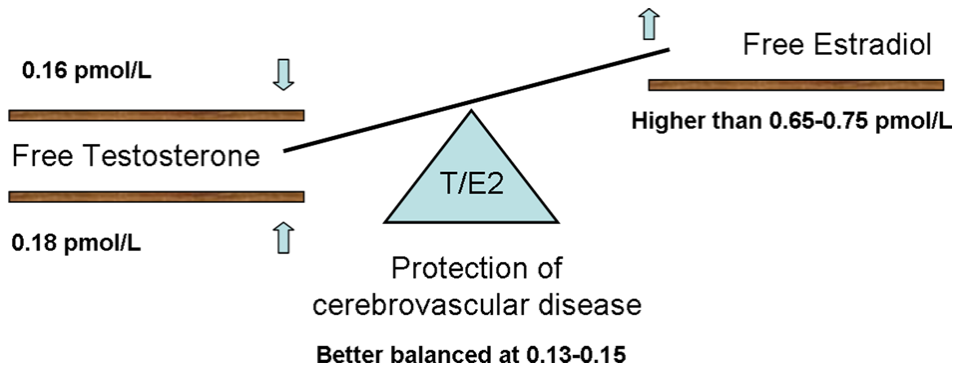
The balance of T/E_2_ in elder men. Better CEVD protection was observed when FE_2_ was higher than 7.5 pmol/L. At the same time, the patient should be kept in the proper ranges of FT and T/E_2_. The balance between T and E_2_ may be more important than their absolute quantities.

Furthermore, in regression with all metabolic continuous variables included, T/E_2_ took the place of FT and FE_2_, with the best CEVD protection having at T/E_2_ ratio of 0.13–0.15, whose OR value was extremely similar to that of FT and FE_2_ (0.23 vs. 0.24–0.25). These values indicated that the T/E_2_ ratio might be the most relevant sex hormone parameter in CEVD. On the other hand, the significant effect of T/E_2_ also reflected the fact that increased SHBG, increased aromatase inhibition, higher TT, lower E_2_ and other factors that change the TT/E_2_ ratio might all contribute to the increased CEVD incidence. The balance between these two hormones may be more important than their absolute quantities, which partly explains why studies focusing on androgens or estrogens alone have produced inconclusive results. For example, postmenopausal women, young men, and polycystic ovary syndrome patients, who have low E_2_ and/or high TT, all have higher risk of vascular diseases than young control women [2]. Therefore, if we want to improve muscle strength or bone BMD in elderly men by testosterone supplementation, a combination of appropriate doses of testosterone and estrogen might be necessary. The proposed balance of T/E_2_ in elderly men is shown in Fig. 4.

Surprisingly, CEVD had a positive correlation with increased FT, and FT, FE_2_ and T/E_2_ all had ideal equations in logistic regression. We believe that including full and comprehensive metabolic indexes and sex hormone indexes in this study was the key difference between our study and other studies. For example, when F/E_2_ was included in the regression, the effect of SHBG disappeared (data not shown). Similarly, the relationships between sex hormones and CEVD were detected only after indexes of metabolism were included, and whether categorical variables or continuous variables were included in the regression affected the results of our analyses. Vlachopoulos et al. [6] also found in a prospective study that the correlation of low plasma T with increased risk of cardiovascular disease in ANOVA was completely reversed in Cox analyses when standard metabolic risk factors were included. We wonder whether analyses based on data with many confounding factors is one of the factors (other than the ratio of T/E_2_) that have confounded the effects of sex hormones on vascular diseases in some former studies.

To put our findings in context, some limitations need to be addressed. Our study is cross-sectional, and the cross-sectional estimates of associations are not true measures of longitudinal changes. We have collected 2000 more subjects in routine physical examinations in 2012, and the 337 subjects of this study have been followed up to the greatest extent possible. An appropriately powered, placebo-controlled trial is being started to verify the possible protective effect of combined supplementation with testosterone and estrogen in elderly men, and the optimal T/E_2_ ratio will also be evaluated.

## Conclusion

Sex hormone levels were tightly correlated with the incidence of cerebrovascular disease in elderly men. Elevated FT levels and a higher T/E_2_ ratio might increase the risk of cerebrovascular disease, while higher FE_2_ decreased this risk. Elevated T/E_2_ was the key parameter in the relationship between sex hormones and the risk of cerebrovascular disease. The balance between T and E_2_ may be more important than their absolute quantities. Extremely low or high T/E_2_ values will harm the brain blood vessels. Careful consideration should be given before beginning testosterone replacement treatment, and supplementation with estrogen seems a good way to protect the blood vessels of the brain in elderly men.

## Supporting Information

Figure S1 **Diagnostic criteria for metabolic syndrome proposed by the Chinese Diabetes Society (CDS).**
(TIF)
