# Supplementary figures and images for: Elevated T/E2 Ratio Is Associated with an Increased Risk of Cerebrovascular Disease in Elderly Men
Source: PLoS One. 2013 Apr 24;8(4):e61598. doi: 10.1371/journal.pone.0061598 (PMC3634802; doi:10.1371/journal.pone.0061598)

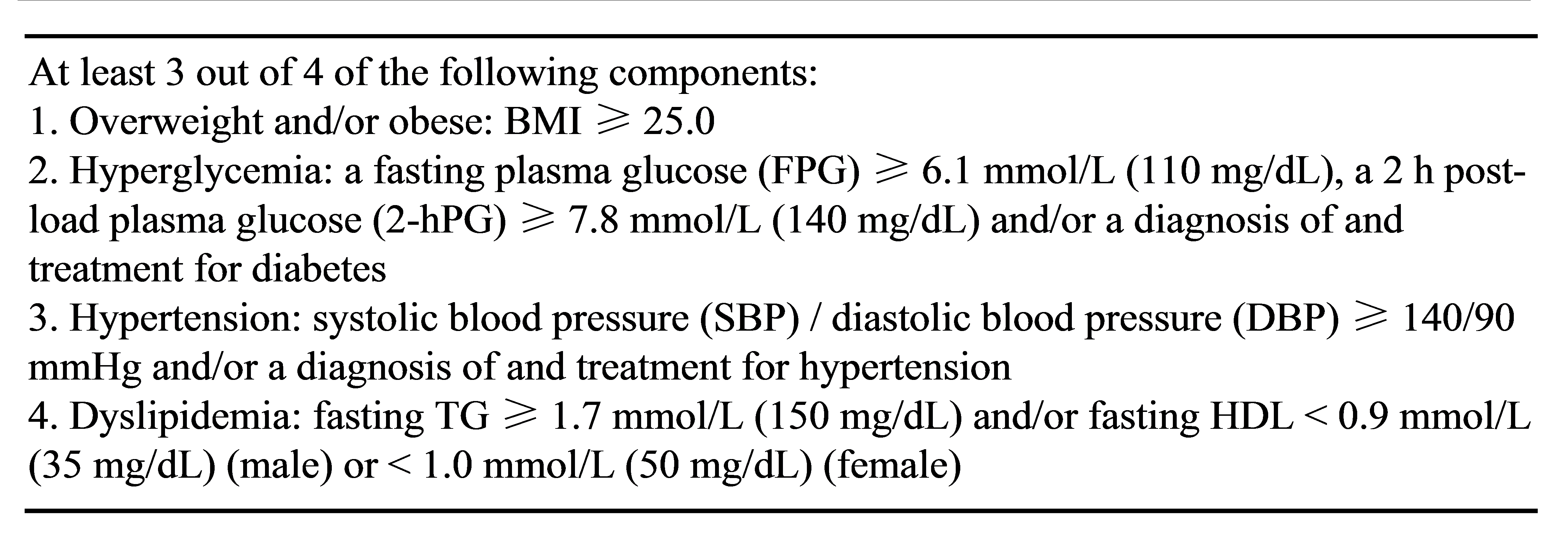

Supplement: Figure S1 — Diagnostic criteria for metabolic syndrome proposed by the Chinese Diabetes Society (CDS). (TIF) [file pone.0061598.s001.tif]
